# COVID-19 Pandemic and Care of Older Adults at Risk for Delirium and Cognitive Vulnerability

**DOI:** 10.5811/westjem.2020.5.47800

**Published:** 2020-06-04

**Authors:** Sangil Lee

**Affiliations:** University of Iowa Carver College of Medicine, Department of Emergency Medicine, Iowa City, Iowa

## BACKGROUND

We are facing a global coronavirus disease 2019 (COVID-19) pandemic since the virus emerged in Wuhan, China. Although this virus does not discriminate on the basis of race, ethnicity, gender, or socioeconomic status, it has the highest mortality rate in older adults. Mentation is an important part of the geriatric evaluation, as it is listed as part of the age-friendly healthcare framework that incorporates four key interventions – what matters; medication; mentation; and mobility (4Ms). Geriatric conditions such as delirium, dementia, and depression will confound an emergent evaluation because of an atypical manifestation of COVID-19 and non-COVID-19 related illness. Furthermore, these conditions are exacerbated by the effects of either social distancing or the financial crisis on vulnerable members of society.^1^ There is a particular concern that delirium will increase amid the COVID-19 pandemic due to the use of infectious disease isolation, and also pose a unique challenge to the evaluation of mentation in older adults, due to both COVID-19 and the common central nervous system (CNS) pathology not related to the COVID-19 such as cerebrovascular accident (CVA).

### COVID-19, Stroke, and Encephalopathy

A recent study showed that the rate of altered consciousness, a cardinal feature of encephalopathy with COVID-19, is about 15%.^2^ As the prevalence of COVID-19 increases, one study found that 30–40% of the COVID-19 patients had non-specific neurological symptoms such as headaches, while more classic and typical symptoms of fever, cough, and dyspnea developed later.^2^ There is a risk of missing CVA if altered mental status is presumed due to COVID-19, since the rate of CVA is 5–6% among the COVID-19 patients.^2^ This study included 214 patients with a mean age of 52.7 years, but there is a paucity of literature focused on older adults who may be at even higher risk due to underlying, more advanced, atherosclerosis.

The mechanism for encephalopathy may be due to an accelerated inflammatory response, the use of sedatives to facilitate mechanical ventilation, and multiple organ dysfunction. In addition, there are reports of meningitis and encephalitis from COVID-19.^3^ The CNS COVID-19 infection occurs through hematogenous spread and also directly via the olfactory nerve. One case report has identified the viral RNA in cerebrospinal fluid even when a nasopharyngeal swab did not detect SARS-CoV-2. A study focused on the geriatric patient population is urgently needed to understand the typical and atypical manifestations of the COVID-19 related CNS pathology.

### The Effect of Social Distancing and Personal Protective Equipment on Cognitively Vulnerable Older Adults

Social distancing measures will likely isolate older adults who live in the community and skilled nursing facilities, which could increase their risk of delirium. As delirium is a response to changes in familiar stimuli in the elder brain, COVID social isolation is an extreme form of absence of stimuli that beget delirium. It is likely that social distancing is an extreme form of catalyst for delirium. The use of personal protective equipment (PPE) and social distancing will likely have a negative effect on patients with dementia and delirium.^4^ Hwang et al, with the Geriatric Emergency Department Collaborative, recently published a one-page handout on delirium in the emergency department that highlights a number of these issues.^5^

Reducing the workforce to minimize the spread of infection will negatively affect the hospital and skilled nursing facility programs that typically help those with or at risk of cognitive impairment. Furthermore, communication, orientation, and early mobilization, which are key to preventing or treating delirium, will be more difficult when providers wear masks, distance themselves, and keep patients in their rooms without visitors. Masks may also introduce barriers for those with hearing impairments who may augment hearing with lip reading. The effects of these isolation precautions may have a negative impact that could trigger or exacerbate delirium or delirium with dementia.

### Evaluation of Older Adults Using the Innovation

The use of telemedicine (tele-stroke) has been presented as an effective modality in the literature.^6^ Telemedicine has been used for diagnostic purposes for sore throat and appendicitis even before the COVID-19 pandemic, but this modality needs to be tested further for delirium evaluation and management. A delirium evaluation includes the confusion assessment method and other tools, and a concise review is available elsewhere.^7^ Delirium assessment over the telephone was conducted almost two decades ago, and study authors concluded that face-to-face assessment was preferred.^8^ Since then, newer tools that are more concise and adaptable with telemedicine have been developed and can be examined in future research.^8^ Hollander et al suggested the optimized use of telehealth visits; it is likely telemedicine will be an effective alternative to in-person visits, decreasing the risk of infection between patients and healthcare providers.^9^ Leveraging telemedicine to diagnose and manage mentation has been implemented in the psychiatry practice amid the COVID pandemic, and there is the potential to improve care for older adults.^10^

## CONCLUSION

The unique aspect of neurological symptoms related to COVID-19 adds to the complexity of emergency preparedness and high-quality care for older adults while minimizing provider exposure. It is recommended that older adults with delirium be routinely evaluated for COVID-19; thus, PPE is necessary to avoid the risk of exposure when COVID-19 infection cannot be ruled out based on prehospital evaluation. The risk of stroke will increase with the COVID-19, and we will need to consider stroke mimics caused by the COVID-19. Telemedicine technology, although potentially disorienting itself, may provide an opportunity to engage, evaluate, and manage older adults more effectively while minimizing in-person exposure for COVID-19.

## References

[1] Fulmer T (2019). Age-friendly health systems transform care for older adults.

[2] Mao LJ, Jin H, Wang M (2020). [Ahead of Print]. Neurologic nanifestations of hospitalized patients with coronavirus disease 2019 in Wuhan, China. JAMA Neurol.

[3] Moriguchi T, Harii N, Goto J (2020). A first case of meningitis/encephalitis associated with SARS-Coronavirus-2. Int J Infect Dis.

[4] Brown EE, Kumar S, Rajji TK (2020). [Ahead of Print]. Anticipating and mitigating the impact of the COVID-19 pandemic on Alzheimer’s disease and related dementias. Am J Geriatr Psychiatry.

[5] Hwang U, Malsch AJ, Biese KJ (2020). Preventing and managing delirium in older emergency department patients during the COVID-19 pandemic. J Geriatr Emerg Med.

[6] Nguyen-Huynh MN, Klingman JG, Avins AL (2018). Novel telestroke program improves thrombolysis for acute stroke across 21 hospitals of an integrated healthcare system. Stroke.

[7] Lee S, Gottlieb M, Mulhausen P (2020). Recognition, prevention, and treatment of delirium in emergency department: an evidence-based narrative review. Am J Emerg Med.

[8] Marcantonio ER, Michaels M, Resnick NM (1998). Diagnosing delirium by telephone. J Gen Intern Med.

[9] Hollander JE, Carr BG (2020). Virtually perfect? Telemedicine for Covid-19. N Engl J Med.

[10] Kalin MG, Garlow SJ, Thertus K (2020). [Ahead of Print]. Thertus K. Rapid implementation of telehealth in hospital psychiatry in response to COVID-19. Am J Psychiatry.

